# Metabolic diversity of human macrophages: potential influence on *Staphylococcus aureus* intracellular survival

**DOI:** 10.1128/iai.00474-23

**Published:** 2024-01-05

**Authors:** Blake P. Bertrand, Dhananjay Shinde, Vinai C. Thomas, Marvin Whiteley, Carolyn B. Ibberson, Tammy Kielian

**Affiliations:** 1 Department of Pathology, Microbiology, and Immunology, University of Nebraska Medical Center, Omaha, Nebraska, USA; 2 School of Biological Sciences, Georgia Institute of Technology, Atlanta, Georgia, USA; 3 School of Biological Sciences, University of Oklahoma, Norman, Oklahoma, USA; St. Jude Children's Research Hospital, Memphis, Tennessee, USA

**Keywords:** *S. aureus*, macrophage, metabolism, intracellular survival, transposon sequencing

## Abstract

*Staphylococcus aureus* is a leading cause of medical device-associated biofilm infections. This is influenced by the ability of *S. aureus* biofilm to evade the host immune response, which is partially driven by the anti-inflammatory cytokine interleukin-10 (IL-10). Here, we show that treatment of human monocyte-derived macrophages (HMDMs) with IL-10 enhanced biofilm formation, suggesting that macrophage anti-inflammatory programming likely plays an important role during the transition from planktonic to biofilm growth. To identify *S. aureus* genes that were important for intracellular survival in HMDMs and how this was affected by IL-10, transposon sequencing was performed. The size of the *S. aureus* essential genome was similar between unstimulated HMDMs and the outgrowth control (18.5% vs 18.4%, respectively, with 54.4% overlap) but increased to 22.5% in IL-10-treated macrophages, suggesting that macrophage polarization status exerts differential pressure on *S. aureus*. Essential genes for *S. aureus* survival within IL-10-polarized HMDMs were dominated by negative regulatory pathways, including nitrogen and RNA metabolism, whereas *S. aureus* essential genes within untreated HMDMs were enriched in biosynthetic pathways such as purine and pyrimidine biosynthesis. To explore how IL-10 altered the macrophage intracellular metabolome, targeted metabolomics was performed on HMDMs from six individual donors. IL-10 treatment led to conserved alterations in distinct metabolites that were increased (dihydroxyacetone phosphate, glyceraldehyde-3-phosphate, and acetyl-CoA) or reduced (fructose-6-phosphate, aspartic acid, and ornithine) across donors, whereas other metabolites were variable. Collectively, these findings highlight an important aspect of population-level heterogeneity in human macrophage responsiveness that should be considered when translating results to a patient population.

**IMPORTANCE:**

One mechanism that *Staphylococcus aureus* biofilm elicits in the host to facilitate infection persistence is the production of the anti-inflammatory cytokine interleukin-10 (IL-10). Here, we show that exposure of human monocyte-derived macrophages (HMDMs) to IL-10 promotes *S. aureus* biofilm formation and programs intracellular bacteria to favor catabolic pathways. Examination of intracellular metabolites in HMDMs revealed heterogeneity between donors that may explain the observed variability in essential genes for *S. aureus* survival based on nutrient availability for bacteria within the intracellular compartment. Collectively, these studies provide novel insights into how IL-10 polarization affects *S. aureus* intracellular survival in HMDMs and the importance of considering macrophage heterogeneity between human donors as a variable when examining effector mechanisms.

## INTRODUCTION

*Staphylococcus aureus* (*S. aureus*) is a Gram-positive bacterium that produces a wide range of virulence factors and has impressive metabolic flexibility. These attributes allow *S. aureus* to occupy a variety of niches within the human body under both physiological and pathological conditions. Regarding the former, up to 30% of the human population is asymptomatically colonized by *S. aureus*, often in the anterior nares (1). However, *S. aureus* can also cause an array of opportunistic infections including endocarditis, pneumonia, skin and soft tissue infection, and prosthetic joint infection (PJI) (2). *S. aureus* also has the propensity to form biofilm at these infection sites, which affords protection from the immune response as well as intrinsic antibiotic tolerance (3).

Macrophages play a critical role in the innate immune response to *S. aureus*, as all tissues within the human body harbor a resident macrophage population (4). Macrophages are professional antigen-presenting cells (APCs) that phagocytose foreign substances and present antigens to the adaptive immune system, in addition to producing cytokines that augment major histocompatibility complex (MHC) expression and drive T cell maturation to potentiate adaptive immunity (5). Furthermore, macrophages produce numerous antimicrobial molecules (i.e., reactive oxygen/nitrogen intermediates and antimicrobial peptides) in response to bacterial challenge (6). However, *S. aureus* expresses many virulence factors to circumvent macrophage-mediated immunity. For example, *S. aureus* can produce several pore-forming toxins including α-hemolysin (Hla), γ-hemolysin (HlgAB and HlgCB), leukocidin ED (LukED), Panton-Valentine leukocidin (PVL or LukSF-PV), and leukocidin AB (LukAB or LukGH) (7, 8). These pore-forming toxins bind to select leukocyte receptors to induce cytotoxicity, which determines their cell-type selectivity (9–12). Species specificity has also been observed for these toxins where HlgAB, HlgCB, PVL, and LukAB, are highly cytolytic for human leukocytes compared to murine cells (10, 13, 14). In addition, several *S. aureus* virulence factors that target the complement pathway or superantigens are selective for human but not mouse leukocytes (15, 16). This demonstrates how well adapted *S. aureus* is to the human host and highlights the importance of utilizing human cells for exploring *S. aureus*-leukocyte interactions (17), an approach that we utilized in the current report.

While the ability of *S. aureus* to survive in the extracellular environment has been well-studied, its ability to occupy an intracellular niche is increasingly recognized as a critical virulence mechanism. *S. aureus* has been shown to survive and replicate within host cells including phagocytes and non-phagocytic cells (18, 19). However, as opposed to other bacteria that subvert phagosome maturation, *S. aureus* survives within mature phagolysosomes and seemingly also requires acidification to trigger intracellular replication (20, 21). Because of this, *S. aureus* must endure the harsh phagolysosomal environment including not only acidification but also reactive oxygen species (ROS) and reactive nitrogen species production, antimicrobial peptides, antimicrobial enzymes, and nutrient sequestration (6). Furthermore, *S. aureus* expresses an array of phenol-soluble modulins that trigger phagosomal escape allowing replication and host cell lysis (22, 23). Other groups have shown that purine biosynthesis is required for intracellular replication within macrophages (24) and *S. aureus* possesses other strategies to survive in this niche (6, 25–27). However, little information is available about how macrophage metabolism shapes *S. aureus* intracellular survival and how this contributes to biofilm formation. Furthermore, how this relationship is regulated in human macrophages and the potential issue of donor variability remains understudied.

Despite the strategies that *S. aureus* uses to circumvent macrophage-mediated immunity, several lines of evidence support that macrophages are important for limiting *S. aureus* pathogenesis. For example, macrophage depletion increases susceptibility to *S. aureus* infection (28, 29). Additionally, the adoptive transfer of proinflammatory polarized macrophages into a mouse model of *S. aureus* biofilm infection promoted bacterial clearance (30), suggesting that the dysfunction of resident macrophages may result from a local inhibitory environment. This is supported by the observation that the anti-inflammatory cytokine interleukin-10 (IL-10) is produced by myeloid cells in response to acute or chronic *S. aureus* infection (31, 32) as well as during nasal colonization (33). IL-10 is a potent mediator that inhibits proinflammatory cytokine production and antigen processing by APCs, including macrophages, which diminishes T cell responsiveness (34, 35).

Furthermore, IL-10 has profound effects on the inflammatory phenotype of macrophages, which is tightly linked to cellular metabolism (36). Following a proinflammatory stimulus, macrophages increase glucose uptake and glycolytic activity concomitant with blocks in the tricarboxylic acid (TCA) cycle. This leads to an accumulation of inflammatory metabolites to augment macrophage antimicrobial activity, including NADPH to induce ROS production, cationic antimicrobial peptides, and itaconate (37). However, anti-inflammatory stimuli such as IL-10 or IL-4 have opposing effects on macrophage metabolism, typified by an intact TCA cycle with increased oxidative phosphorylation (OxPhos) and fatty acid oxidation (37). Therefore, altering macrophage metabolism is a potential mechanism to reverse the anti-inflammatory properties of resident macrophages following *S. aureus* exposure. Indeed, we have shown that resident macrophages and monocytes infiltrating the site of PJI exhibited an oxidative bias, and metabolic reprogramming with the OxPhos inhibitor oligomycin transformed these cells toward a proinflammatory state, which was conducive to bacterial clearance (38). A recent study showed that IL-4-treated macrophages exhibited increased *S. aureus* burden and favored small colony variant phenotypes (39), suggesting that macrophage polarization impacts *S. aureus* intracellular survival.

Here, we demonstrate that *S. aureus* exploits IL-10 polarized human monocyte-derived macrophages (HMDMs) to promote its intracellular survival and biofilm development. We next employed transposon sequencing (Tn-seq) to understand the strategies that *S. aureus* uses for intracellular survival within IL-10-polarized HMDMs, along with metabolomics to discover how IL-10 shapes the intracellular metabolome while addressing the issue of human variability in these metabolic profiles. These findings revealed marked donor variability in metabolic responses that may influence the ability of *S. aureus* to survive in human macrophages.

## RESULTS

### Exposure of human macrophages to IL-10 enhances biofilm formation

**Fig 1:**
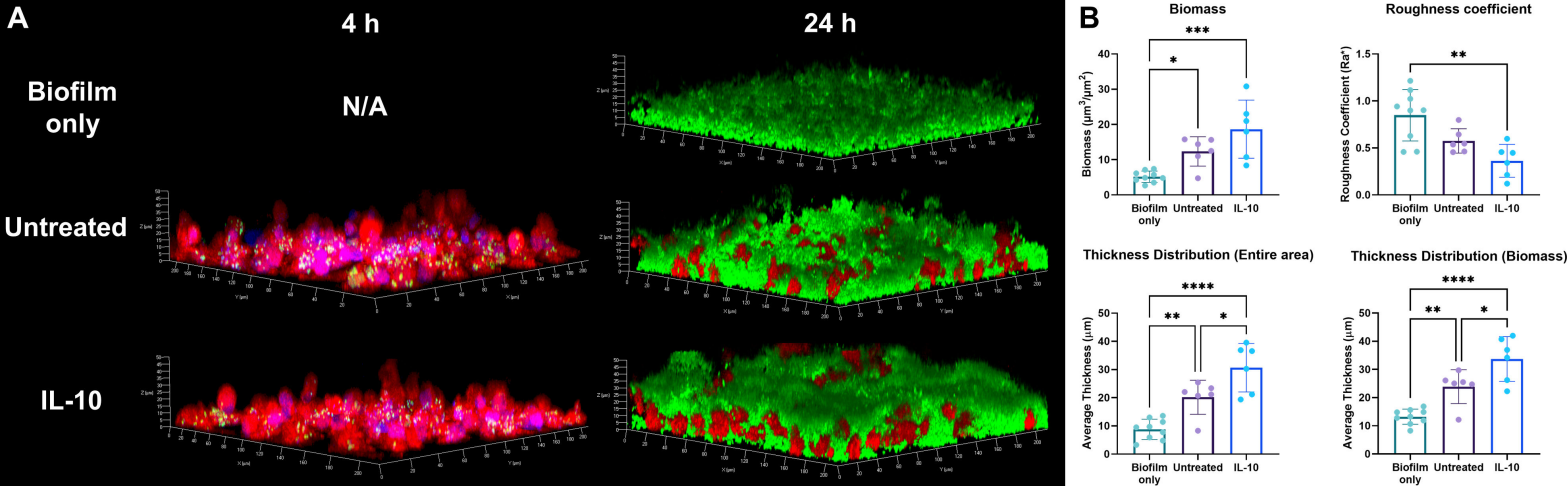
IL-10 treatment of HMDMs enhances biofilm formation. (**A**) HMDMs were untreated or exposed to IL-10 for 16 h, stained with CellTracker Deep Red (red) and Hoechst 33342 (blue), then challenged with live *S. aureus*-tdTomato (pseudo-colored green) at an MOI of 10:1 (bacteria:macrophage). Z-stacks (1 µm) were captured at the indicated times by confocal microscopy and subjected to 3D rendering and (**B**) biofilms were quantified by Comstat2 (*n* = 6 biological replicates from two independent experiments). Biofilms without HMDMs were grown on separate days and subjected to Comstat2 measurements (*n* = 9 biological replicates from two independent experiments). *P < 0.05; **P < 0.01; ***P < 0.001; and ****P < 0.0001.

Resident tissue macrophages are the first to encounter invading bacteria and recent studies have shown that *S. aureus* can survive intracellularly in these cells (20, 40). Although our prior work has revealed an important role for IL-10 in promoting the persistence of mature *S. aureus* biofilm (32, 41), it remains unknown whether IL-10 accelerates biofilm development and how macrophages influence this process. To address this question, primary HMDMs were treated with IL-10 or vehicle for 16 h prior to challenge with live *S. aureus*-GFP (USA300, LAC), where human cells were used to enhance the translational impact of our findings. Nearly all bacteria were phagocytosed after a 4-h co-culture period, with no discernable differences detected between vehicle or IL-10-treated macrophages (Fig. 1A). Live cell imaging demonstrated *S. aureus* replication within HMDMs from each treatment condition, followed by macrophage lysis and release of bacteria into the extracellular space (Video S1). By 24 h, biofilm formation was evident with bacteria surrounding and covering macrophages (Fig. 1A). Interestingly, IL-10-treated HMDMs augmented biofilm development, as evident by a significantly thicker biofilm and more tower-like structures at 24 h compared to untreated macrophages and *S. aureus* only (Fig. 1A and B). In addition, many IL-10-treated HMDMs were embedded within the biofilm, whereas most vehicle-treated macrophages remained exposed at the biofilm surface. In general, HMDMs promoted biofilm formation, as revealed by significantly increased biomass and thickness compared to *S. aureus* only (Fig. 1B). Interestingly, no significant differences in *S. aureus* intracellular survival were observed between untreated and IL-10-treated HMDMs over a 24-h period (Fig. S1A). Furthermore, this was observed for several *S. aureus* strains including JE-2 (USA300, CC8), UAMS-1 (USA200, CC30), MW2 (USA400, CC1), and SA564 (USA100, CC5) after a 24-h incubation period (Fig. S1B).

### Polarization of human macrophages with IL-10 expands the essential genome of *S. aureus* for intracellular survival

**Fig 2:**
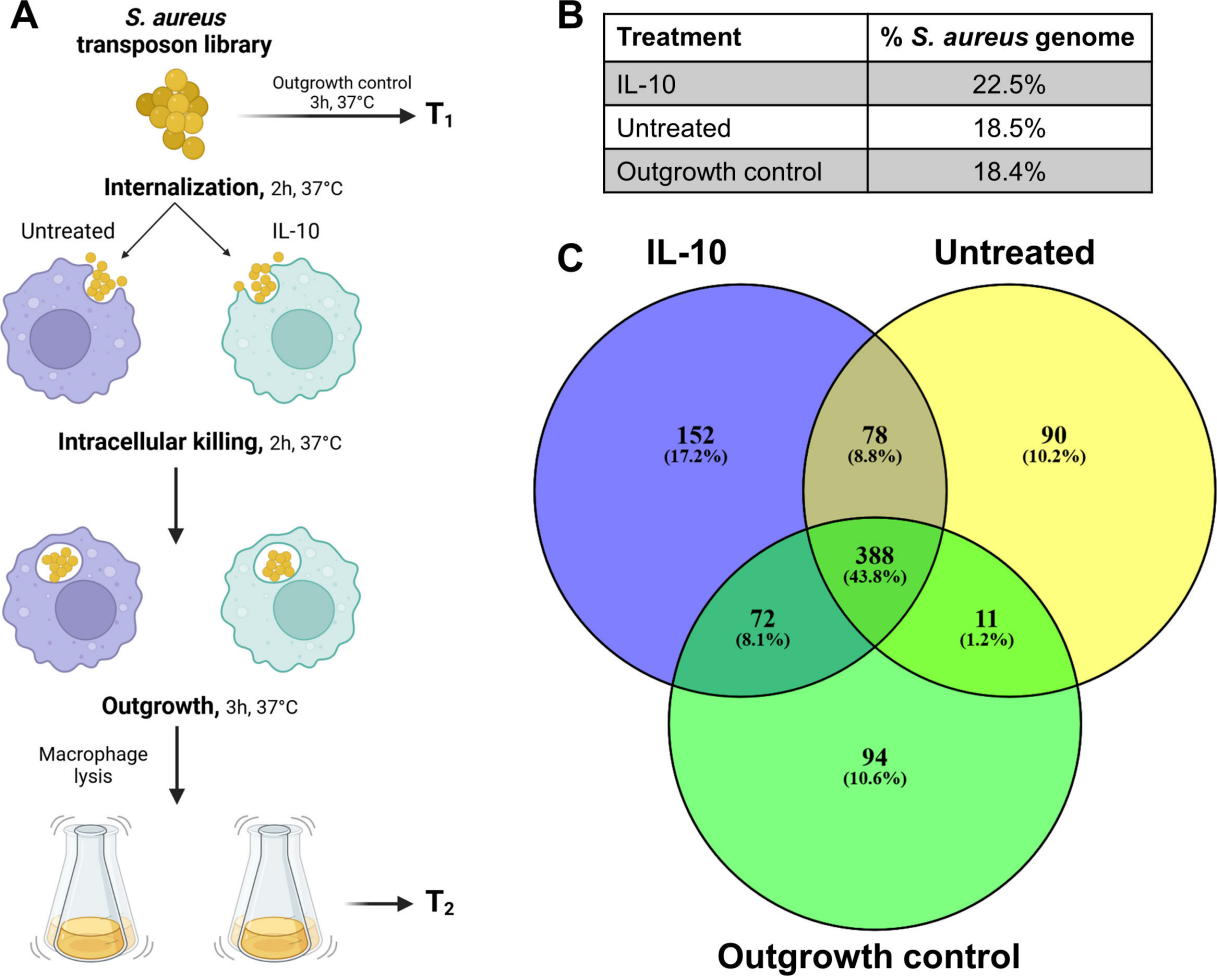
The essential genome of *S. aureus* is expanded in IL-10-treated HMDMs. (**A**) Schematic of the methodology used for Tn-seq. HMDMs were untreated or exposed to IL-10 for 16 h and then inoculated with a saturated *S. aureus* Tn library at an MOI of 10:1 (bacteria:macrophage). HMDMs were treated with a high dose of gentamicin after a 2-h phagocytosis period to kill remaining extracellular *S. aureus* and then transitioned to a low gentamicin dose to prevent intracellular accumulation of antibiotic. HMDMs were then lysed to recover viable intracellular bacteria, which were subjected to a 3-h outgrowth period alongside an aliquot of the same Tn library as an outgrowth control. Figure was created using BioRender. (**B and C**) Essential genes from each condition (IL-10, untreated, or outgrowth control) were identified using a previously established Monte-Carlo method (*P* < 0.01) and are represented as (**B**) a percentage of the *S. aureus* genome or (**C**) a Venn diagram.

To determine the relative fitness contribution for *S. aureus* intracellular survival within macrophages and how this was influenced by IL-10, a Tn-seq approach was utilized. First, to validate that Tn-seq would be sensitive enough to discern differences in *S. aureus* intracellular survival, gentamicin protection assays were performed (42) using mutants with well-described defects in virulence factor production, namely *sigB*, *agrA*, *saeR*, and *hla* (43–46). This approach confirmed that all *S. aureus* mutants had decreased intracellular burden in HMDMs (Fig. S2) as has been previously reported (40, 47). For Tn-seq, HMDMs were treated with IL-10 or vehicle for 16 h prior to inoculation with a saturated Tn library in *S. aureus* LAC (48). To ensure that enough bacteria reached the intracellular compartment to achieve adequate coverage of the Tn library and prevent downstream bottlenecks, the phagocytosis period was extended to 2 h, and HMDMs were incubated for an additional 2 h after gentamicin treatment to provide sufficient time for mutants to be subjected to pressures within the intracellular compartment. HMDMs were then lysed, and the resulting bacteria were outgrown for 3 h along with an outgrowth control of the initial Tn library for the same interval (Fig. 2A). The 3-h outgrowth period was selected to increase bacterial density for Tn-seq, but importantly, it was shorter than prior studies (49) to minimize potential biases from extended growth in a nutrient-rich medium. *S. aureus* essential genes from each condition (outgrowth control, untreated HMDM, and IL-10-treated HMDM) were determined using a Monte-Carlo simulation-based method to compare the reads mapping to each gene against 400 pseudo data sets generated by randomly distributing reads across possible Tn insertion sites within the *S. aureus* genome as previously described (50, 51).

**Fig 3:**
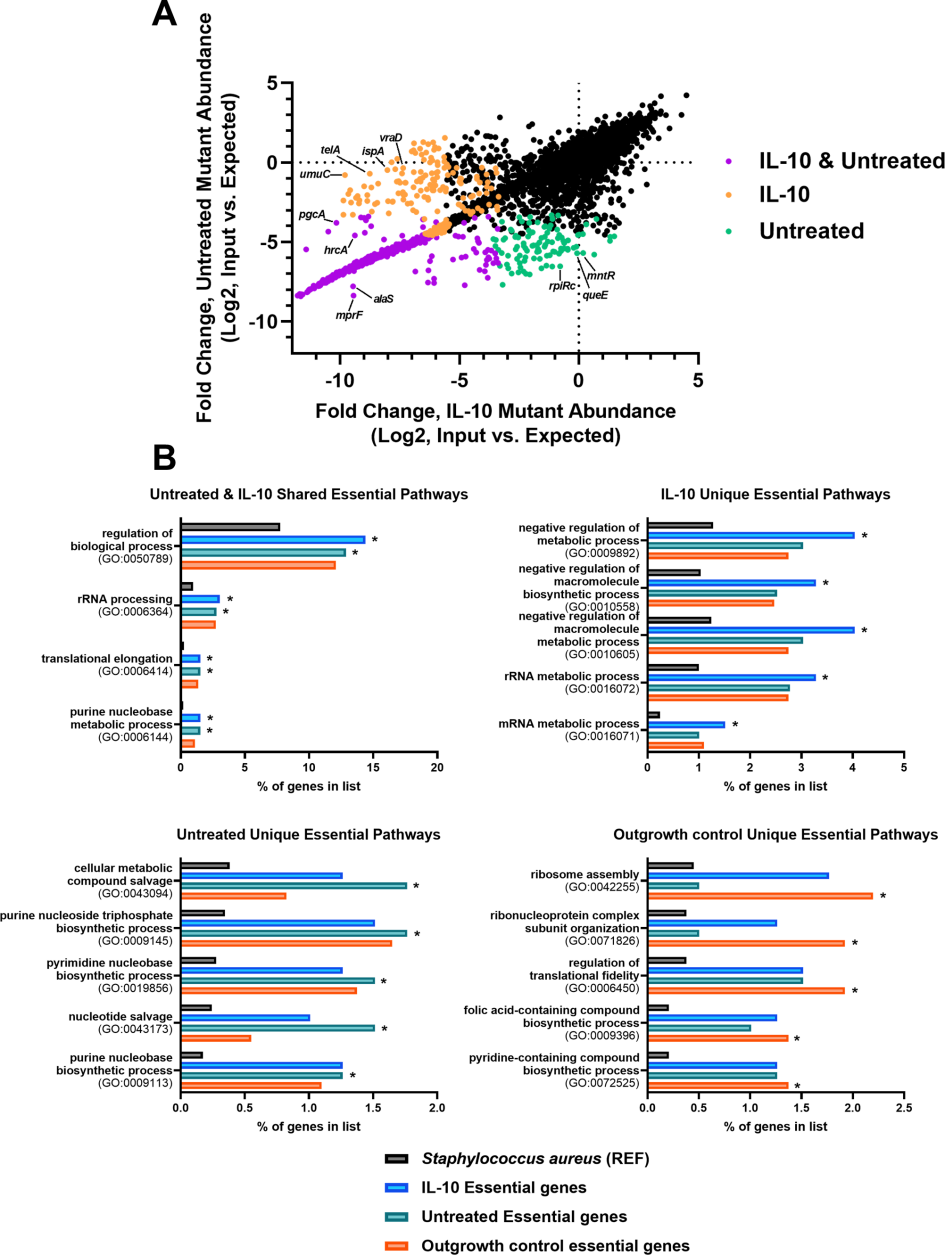
Tn-seq identifies pathways important for *S. aureus* intracellular survival in HMDMs depending on polarization status. (**A**) Volcano plot showing the abundance of *S. aureus* mutants within IL-10-treated or untreated HMDMs with black dots depicting genes that were non-essential in either condition. (**B**) Essential pathways in each treatment condition were determined from gene lists by a statistical overrepresentation test using PANTHER GO Biological Process Complete (FDR-adjusted *P*-value < 0.01; Fisher’s Exact test). REF, reference genome.

Treatment of HMDMs with IL-10 expanded the essential genome of *S. aureus*, as 22.5% of the genome was required for intracellular survival compared to 18.5% and 18.4% for untreated HMDMs and the outgrowth control, respectively (Fig. 2B). Over 70% of the genes that were essential in the outgrowth control were also classified as essential in previously published data sets (Fig. S3) (48, 49), suggesting that our 3-h outgrowth period had minimal effects on the essentiality of the genome. We identified 152 genes that were essential for survival only in IL-10-treated HMDMs, 90 genes that were essential only in untreated HMDMs, and 78 genes that were essential in both conditions (Fig. 2C). Among the top 10 essential genes that were unique to IL-10-treated HMDMs were *ilvD* (SAUSA300_RS11035), *gudB* (SAUSA300_RS04645), *hemX* (SAUSA300_RS08820), *miaA* (SAUSA300_RS06455), and *ndh2* (SAUSA300_RS04560) (Fig. 3A; Table 1). In terms of untreated HMDMs, unique genes included *itaA* (SAUSA300_RS04930), *pckA* (SAUSA300_RS09470), *katA* (SAUSA300_RS06680), *aspB* (SAUSA300_RS10490), and *bshA* (SAUSA300_RS07355) (Fig. 3A; Table 1). Many essential genes were shared between IL-10 and untreated HMDMs, which included *polA* (SAUSA300_RS08925), *pgcA* (SAUSA300_RS13475), *atpD* (SAUSA300_RS11335), *atpA* (SAUSA300_RS11345), *mprF* (SAUSA300_RS06820), and *sucB* (SAUSA300_RS07100) (Fig. 3A; Table 1). The full list of essential genes for each condition is presented in Data Set S1.

**TABLE 1:** Top 10 *S*. *aureus* essential genes for survival within untreated or IL-10-treated HMDMs as identified by Tn-seq

		Untreated			IL-10			Outgrowth		
	*S. aureus*— REFLIST (2,889)	Essential genes (396)	Fold enrichment	*q*-value	Essential genes (439)	Fold enrichment	*q*-value	Essential genes (364)	Fold enrichment	*q*-value
Untreated unique essential pathways										
Purine nucleobase biosynthetic process (GO:0009113)	5	5	7.3	0.040*	5	6.58	0.060	4	6.35	0.105
Nucleotide salvage (GO:0043173)	7	6	6.25	0.026*	4	3.76	0.301	2	2.27	0.758
Pyrimidine nucleobase biosynthetic process (GO:0019856)	8	6	5.47	0.040*	5	4.11	0.164	5	4.96	0.092
Purine nucleoside triphosphate biosynthetic process (GO:0009145)	10	7	5.11	0.026*	6	3.95	0.116	6	4.76	0.057
Cellular metabolic compound salvage (GO:0043094)	11	7	4.64	0.037*	5	2.99	0.308	3	2.16	0.793
DNA duplex unwinding (GO:0032508)	15	8	3.89	0.041*	7	3.07	0.160	7	3.7	0.073
RNA phosphodiester bond hydrolysis (GO:0090501)	18	9	3.65	0.034*	9	3.29	0.061	7	3.09	0.136
Tetrapyrrole metabolic process (GO:0033013)	19	9	3.46	0.043*	8	2.77	0.160	8	3.34	0.071
RNA modification (GO:0009451)	47	17	2.64	0.016*	13	1.82	0.317	11	1.86	0.464
Cellular response to DNA damage stimulus (GO:0006974)	57	19	2.43	0.021*	14	1.62	0.510	10	1.39	0.836
Cellular response to stress (GO:0033554)	77	24	2.27	0.011*	19	1.62	0.361	14	1.44	0.671
DNA metabolic process (GO:0006259)	121	32	1.93	0.016*	27	1.47	0.432	20	1.31	0.772
Cellular response to stimulus (GO:0051716)	132	34	1.88	0.016*	28	1.4	0.544	18	1.08	1.000
Response to stress (GO:0006950)	117	30	1.87	0.030*	24	1.35	0.650	18	1.22	0.949
Response to stimulus (GO:0050896)	199	47	1.72	0.013*	41	1.36	0.409	28	1.12	1.000
IL-10 unique essential pathways										
mRNA metabolic process (GO:0016071)	7	4	4.17	0.212	6	5.64	0.044*	4	4.54	0.195
rRNA metabolic process (GO:0016072)	29	11	2.77	0.058	13	2.95	0.029*	10	2.74	0.079
Negative regulation of macromolecule metabolic process (GO:0010605)	36	12	2.43	0.101	16	2.92	0.011*	10	2.2	0.228
Negative regulation of macromolecule biosynthetic process (GO:0010558)	30	10	2.43	0.168	13	2.85	0.033*	9	2.38	0.247
Negative regulation of metabolic process (GO:0009892)	37	12	2.37	0.111	16	2.85	0.020*	10	2.15	0.236
Negative regulation of biological process (GO:0048519)	39	12	2.24	0.131	16	2.7	0.025*	10	2.04	0.377
Untreated and IL-10 shared essential pathways										
Purine nucleobase metabolic process (GO:0006144)	6	6	7.3	0.017*	6	6.58	0.031*	4	5.29	0.152
Translational elongation (GO:0006414)	7	6	6.25	0.027*	6	5.64	0.044*	5	5.67	0.067
rRNA processing (GO:0006364)	28	11	2.87	0.050*	12	2.82	0.045*	10	2.83	0.069
Regulation of biological process (GO:0050789)	224	51	1.66	0.012*	57	1.67	0.009*	44	1.56	0.061
Outgrowth unique essential pathways										
Pyridine-containing compound biosynthetic process (GO:0072525)	6	5	6.08	0.059	5	5.48	0.089	5	6.61	0.045*
Folic acid-containing compound biosynthetic process (GO:0009396)	6	4	4.86	0.173	5	5.48	0.089	5	6.61	0.045*
Regulation of translational fidelity (GO:0006450)	11	6	3.98	0.101	6	3.59	0.152	7	5.05	0.025*
Ribonucleoprotein complex subunit organization (GO:0071826)	11	2	1.33	1.000	5	2.99	0.309	7	5.05	0.025*
Ribosome assembly (GO:0042255)	13	2	1.12	1.000	7	3.54	0.105	8	4.88	0.015*
RNA biosynthetic process (GO:0032774)	20	9	3.28	0.054	8	2.63	0.186	11	4.37	0.005*
Organelle assembly (GO:0070925)	15	2	0.97	1.000	7	3.07	0.158	8	4.23	0.027*
Purine nucleoside diphosphate metabolic process (GO:0009135)	16	7	3.19	0.126	8	3.29	0.088	8	3.97	0.035*
DNA-templated transcription (GO:0006351)	16	6	2.74	0.243	5	2.06	0.686	8	3.97	0.034*
Protein-containing complex organization (GO:0043933)	23	6	1.9	0.588	10	2.86	0.079	11	3.8	0.010*
Protein-containing complex assembly (GO:0065003)	19	4	1.54	0.756	8	2.77	0.159	9	3.76	0.027*
Fatty acid metabolic process (GO:0006631)	29	11	2.77	0.059	10	2.27	0.220	13	3.56	0.006*

To gain broader insights into systems that *S. aureus* uses to survive within HMDMs, pathway analysis was performed on each of the unique gene lists using over-representation analysis (Fisher’s exact test, FDR-adjusted *P* < 0.01). Essential Gene Ontology (GO) pathways for *S. aureus* survival in IL-10-treated HMDMs were related to mRNA/rRNA metabolic process (GO:0016071/0016072) and the negative regulation of several biosynthetic pathways, including metabolic process (GO:0009892), macromolecule biosynthetic process (GO:0010558), and macromolecule metabolic process (GO:0010605) (Fig. 3B; Table 2).

**TABLE 2:** Significant *S. aureus* pathways for survival within untreated and/or IL-10-treated HMDMs^*a*^

		Untreated			IL-10			Outgrowth		
	*S. aureus—* REFLIST (2,889)	Essential genes (396)	Fold enrichment	*q*-value	Essential genes (439)	Fold enrichment	*q*-value	Essential genes (364)	Fold enrichment	*q*-value
Untreated unique essential pathways										
Purine nucleobase biosynthetic process (GO:0009113)	5	5	7.3	0.040*	5	6.58	0.060	4	6.35	0.105
Nucleotide salvage (GO:0043173)	7	6	6.25	0.026*	4	3.76	0.301	2	2.27	0.758
Pyrimidine nucleobase biosynthetic process (GO:0019856)	8	6	5.47	0.040*	5	4.11	0.164	5	4.96	0.092
Purine nucleoside triphosphate biosynthetic process (GO:0009145)	10	7	5.11	0.026*	6	3.95	0.116	6	4.76	0.057
Cellular metabolic compound salvage (GO:0043094)	11	7	4.64	0.037*	5	2.99	0.308	3	2.16	0.793
DNA duplex unwinding (GO:0032508)	15	8	3.89	0.041*	7	3.07	0.160	7	3.7	0.073
RNA phosphodiester bond hydrolysis (GO:0090501)	18	9	3.65	0.034*	9	3.29	0.061	7	3.09	0.136
Tetrapyrrole metabolic process (GO:0033013)	19	9	3.46	0.043*	8	2.77	0.160	8	3.34	0.071
RNA modification (GO:0009451)	47	17	2.64	0.016*	13	1.82	0.317	11	1.86	0.464
Cellular response to DNA damage stimulus (GO:0006974)	57	19	2.43	0.021*	14	1.62	0.510	10	1.39	0.836
Cellular response to stress (GO:0033554)	77	24	2.27	0.011*	19	1.62	0.361	14	1.44	0.671
DNA metabolic process (GO:0006259)	121	32	1.93	0.016*	27	1.47	0.432	20	1.31	0.772
Cellular response to stimulus (GO:0051716)	132	34	1.88	0.016*	28	1.4	0.544	18	1.08	1.000
Response to stress (GO:0006950)	117	30	1.87	0.030*	24	1.35	0.650	18	1.22	0.949
Response to stimulus (GO:0050896)	199	47	1.72	0.013*	41	1.36	0.409	28	1.12	1.000
IL-10 unique essential pathways										
mRNA metabolic process (GO:0016071)	7	4	4.17	0.212	6	5.64	0.044*	4	4.54	0.195
rRNA metabolic process (GO:0016072)	29	11	2.77	0.058	13	2.95	0.029*	10	2.74	0.079
Negative regulation of macromolecule metabolic process (GO:0010605)	36	12	2.43	0.101	16	2.92	0.011*	10	2.2	0.228
Negative regulation of macromolecule biosynthetic process (GO:0010558)	30	10	2.43	0.168	13	2.85	0.033*	9	2.38	0.247
Negative regulation of metabolic process (GO:0009892)	37	12	2.37	0.111	16	2.85	0.020*	10	2.15	0.236
Negative regulation of biological process (GO:0048519)	39	12	2.24	0.131	16	2.7	0.025*	10	2.04	0.377
Untreated and IL-10 shared essential pathways										
Purine nucleobase metabolic process (GO:0006144)	6	6	7.3	0.017*	6	6.58	0.031*	4	5.29	0.152
Translational elongation (GO:0006414)	7	6	6.25	0.027*	6	5.64	0.044*	5	5.67	0.067
rRNA processing (GO:0006364)	28	11	2.87	0.050*	12	2.82	0.045*	10	2.83	0.069
Regulation of biological process (GO:0050789)	224	51	1.66	0.012*	57	1.67	0.009*	44	1.56	0.061
Outgrowth unique essential pathways										
Pyridine-containing compound biosynthetic process (GO:0072525)	6	5	6.08	0.059	5	5.48	0.089	5	6.61	0.045*
Folic acid-containing compound biosynthetic process (GO:0009396)	6	4	4.86	0.173	5	5.48	0.089	5	6.61	0.045*
Regulation of translational fidelity (GO:0006450)	11	6	3.98	0.101	6	3.59	0.152	7	5.05	0.025*
Ribonucleoprotein complex subunit organization (GO:0071826)	11	2	1.33	1.000	5	2.99	0.309	7	5.05	0.025*
Ribosome assembly (GO:0042255)	13	2	1.12	1.000	7	3.54	0.105	8	4.88	0.015*
RNA biosynthetic process (GO:0032774)	20	9	3.28	0.054	8	2.63	0.186	11	4.37	0.005*
Organelle assembly (GO:0070925)	15	2	0.97	1.000	7	3.07	0.158	8	4.23	0.027*
Purine nucleoside diphosphate metabolic process (GO:0009135)	16	7	3.19	0.126	8	3.29	0.088	8	3.97	0.035*
DNA-templated transcription (GO:0006351)	16	6	2.74	0.243	5	2.06	0.686	8	3.97	0.034*
Protein-containing complex organization (GO:0043933)	23	6	1.9	0.588	10	2.86	0.079	11	3.8	0.010*
Protein-containing complex assembly (GO:0065003)	19	4	1.54	0.756	8	2.77	0.159	9	3.76	0.027*
Fatty acid metabolic process (GO:0006631)	29	11	2.77	0.059	10	2.27	0.220	13	3.56	0.006*

^*a*^ A statistical overrepresentation test using *S. aureus* essential gene lists from untreated or IL-10-treated HMDMs, shared between untreated and IL-10-treated HMDMs, and outgrowth control was performed in PANTHER using PANTHER GO Biological Process Complete pathways (FDR-adjusted *P*-value < 0.01; Fisher’s exact test). REFLIST, *S. aureus* reference gene list.

In contrast, pathways enriched for survival in untreated HMDMs were largely biosynthetic and included cellular metabolic compound salvage (GO:0043094), purine nucleoside triphosphate biosynthetic process (GO:0009145), pyrimidine nucleobase biosynthetic process (GO:0019856), nucleotide salvage (GO:0043173), and purine nucleobase biosynthetic process (GO:0009113) (Fig. 3B; Table 2). Several essential pathways for *S. aureus* intracellular survival were shared between untreated and IL-10-treated HMDMs such as regulation of biological process (GO:0050789) and rRNA processing (GO:0006364) (Fig. 3B; Table 2). Taken together, these results suggest that HMDMs force the essentiality of a core set of *S. aureus* genes for intracellular survival regardless of their activation state; however, IL-10 polarization seems to involve additional pressures to force the essentiality of a unique *S. aureus* gene set that is biased for catabolism.

### *S. aureus* MprF is critical for intracellular survival within HMDMs

**Fig 4:**
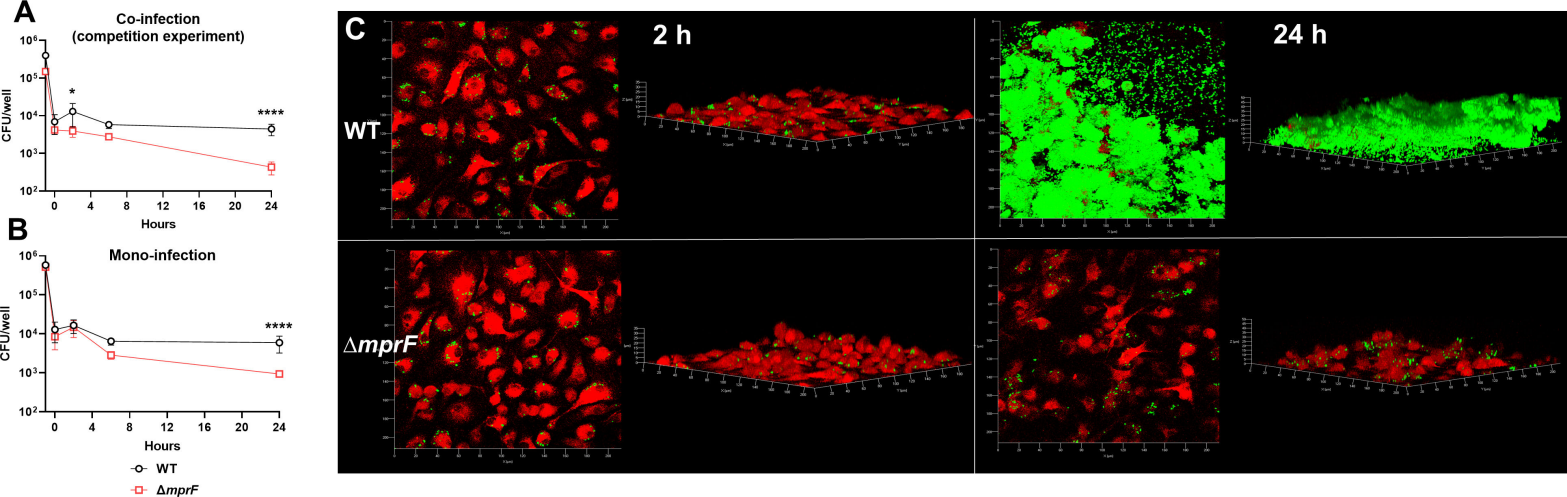
*S. aureus* MprF is critical for intracellular survival in HMDMs and biofilm formation. HMDMs were exposed to *S. aureus* WT or ∆*mprF* under (**A**) competition or (**B**) mono-infection conditions and lysed to quantify intracellular bacterial burden at the indicated time points using a gentamicin protection assay (*n* = 4 from one experiment; *P < 0.05 and ****P < 0.0001; two-way ANOVA with Sidak’s multiple comparisons test). (**C**) To determine the effect of intracellular survival on biofilm formation, HMDMs were stained with CellTracker Deep Red (red) and then infected with either GFP-expressing *S. aureus* WT pCM29 or ∆*mprF* pCM29 (green). Z-stacks (1 µM) were acquired using confocal microscopy at 2- or 24-h post-infection and 3D rendering was performed.

To validate a subset of our Tn-seq findings, we first examined genes that were essential for survival in both untreated and IL-10-treated HMDMs. One of these genes, *mprF*, encodes for the *S. aureus* multiple peptide resistance factor (MprF), which has been shown to play a role in resistance to cationic antimicrobial peptides (CAMPs) by modifying negatively charged membrane phosphatidylglycerol with positively charged lysine residues (52). This modification reduces the affinity of CAMPs for the bacterial membrane (52), and *mprF* has been shown to be induced within the macrophage phagolysosome and contribute to *S. aureus* survival in macrophages (25). Our findings validated the role of MprF for intracellular survival within HMDMs under both competition (Fig. 4A) and mono-infection (Fig. 4B) conditions. We leveraged the divergent intracellular survival properties of the *mprF* mutant to explore how HMDMs may serve as a nucleation source to promote biofilm development. Both wild-type (WT) and ∆*mprF* were phagocytosed by HMDMs to roughly equivalent extents within 2 h (Fig. 4C); however, by 24 h, WT bacteria had escaped from HMDMs to establish a robust biofilm, whereas biofilm formation was absent with the *mprF* mutant although bacteria were still visible (Fig. 4C).

After validating the role of *mprF* in promoting *S. aureus* survival in HMDMs and biofilm formation, we sought to characterize other Tn-seq hits. We have previously shown that *S. aureus atpA* and *atpG* are important for survival within macrophages (53), which were identified in this Tn-seq screen. We selected additional mutants that were essential for survival within IL-10-treated HMDMs based on pathway analysis including *pgcA* (SAUSA300_RS13475), *hrcA* (SAUSA300_RS08405), *cvfB* (SAUSA300_RS06980), and *phoH* (SAUSA300_RS08350). However, besides *mprF*, no mutants displayed significant decreases in survival within HMDMs (Fig. S4). While Tn-seq is prone to false positives (54), there is also the possibility that human monocyte donor variability may contribute to differences in bacterial persistence and IL-10 responsiveness in HMDMs, which is supported by the finding that survival of WT bacteria varied by 1.5 logs (Fig. S4). This became a critical issue to address since our Tn-seq study was performed with HMDMs from one donor and hits could conceivably be influenced by donor-dependent differences.

### Donor variability leads to divergence in the HMDM metabolome

**Fig 5:**
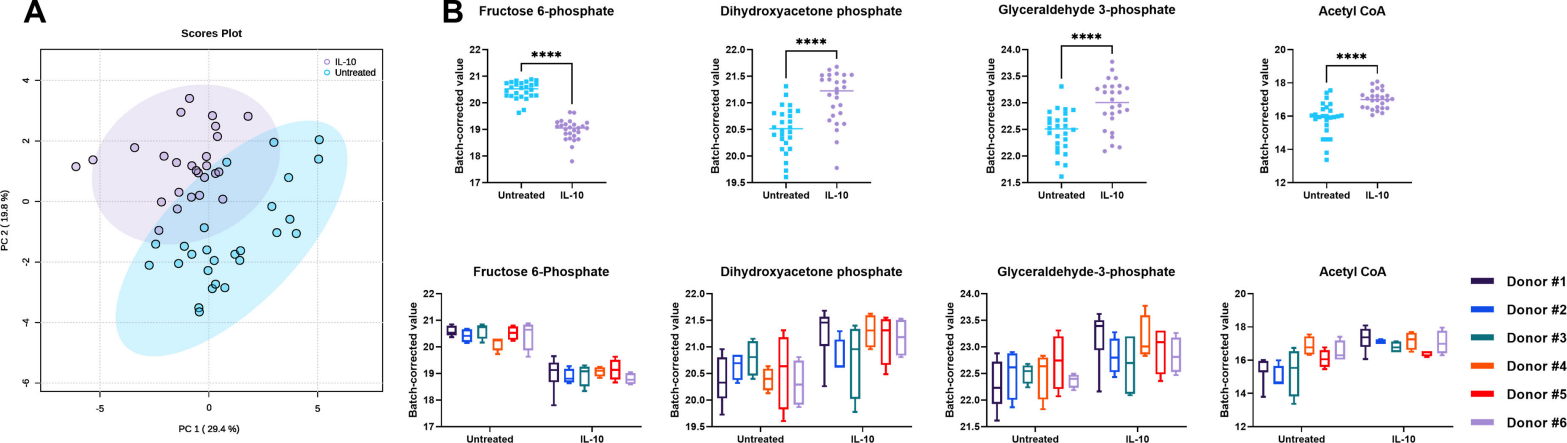
IL-10 elicits a conserved central carbon metabolic response in HMDMs. HMDMs were untreated or exposed to IL-10 for 16 h, whereupon intracellular metabolites were analyzed using LC-MS/MS. (**A**) Principal component analysis and (**B**) changes in metabolites from central carbon metabolism across all HMDM donors. (*n* = 4–6 biological replicates for each of the six healthy human donors; ****P < 0.0001; Student’s *t*-test). Changes were validated using SAM (significance analysis of metabolites) and EBAM (empirical Bayesian analysis of metabolites) in Metaboanalyst.

It is well appreciated that *S. aureus* growth is dictated by nutrient availability, and the intracellular milieu represents a unique metabolic landscape for bacteria to adapt (55–57). To explore how donor variability may impact HMDM responsiveness and potentially *S. aureus* survival, intracellular metabolites in IL-10 or untreated HMDMs from six different healthy human donors were quantified using targeted liquid chromatography (LC) -tandem mass spectrometry (MS)/MS. Data were subjected to batch correction to allow for comparisons between samples that were prepared and analyzed on separate days (58, 59). Principal component analysis (PCA) revealed that IL-10 elicited a distinct metabolic signature in HMDMs as evident by their separate clustering from untreated HMDMs across donors (Fig. 5A). IL-10 exposure led to significant alterations in several metabolites involved in central carbon metabolism including decreased fructose 6-phosphate and increased dihydroxyacetone phosphate, glyceraldehyde 3-phosphate, and acetyl CoA compared to untreated HMDMs, reflecting a core response to IL-10 across donors (Fig. 5B). Interestingly, the metabolic response to IL-10 was still evident when HMDMs were challenged with heat-killed *S. aureus* although less resolved from untreated HMDMs (Fig. S5), suggesting that IL-10 treatment leaves a footprint on HMDMs even after exposure to a proinflammatory stimulus.

**Fig 6:**
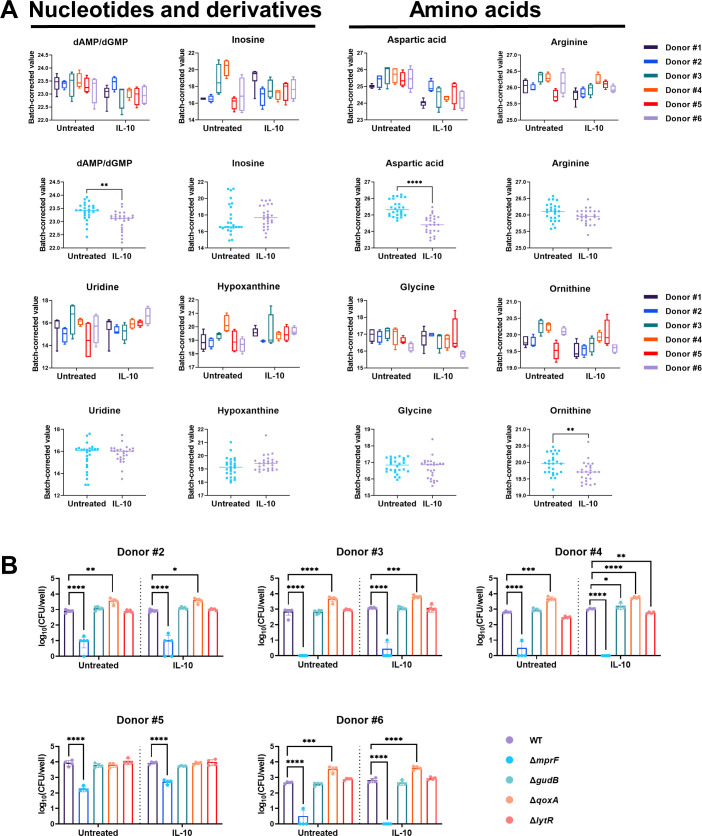
Human donor variability affects select metabolites in HMDMs. (**A**) The intracellular metabolites of HMDMs from six different healthy human donors were analyzed by LC-MS/MS with nucleotides, nucleotide derivatives, and amino acids reported (*n* = 4–6 biological replicates for each of the six independent healthy donors; **P < 0.01 and ****P < 0.0001). The top graphs show metabolites split by donor, and the bottom graphs depict the data from each donor combined by treatment group. (**B**) Gentamicin protection assays were performed on HMDMs from five of the same donors to analyze the killing capacity of wild-type *S. aureus* vs various mutants (*n* = 4 biological replicates from each of the five independent donors; *P < 0.05; **P < 0.01; ***P < 0.001; and ****P < 0.0001; one-way ANOVA with a Dunnett’s multiple comparisons test against WT for each condition).

While our findings revealed that some metabolic intermediates of glycolysis and the TCA cycle were concordantly regulated in HMDMs, other metabolites exhibited changes that were donor dependent. For example, nucleotide and amino acid abundance in both untreated and IL-10-treated HMDMs from different donors were variable (Fig. 6A; Data Set S2). Nevertheless, in aggregate, significant reductions in dAMP/dGMP, aspartic acid, and ornithine were observed following IL-10 exposure (Fig. 6A). These metabolic fluctuations between donors may affect nutrient availability within HMDMs for intracellular *S. aureus* and thus change the essentiality of corresponding genes, which act as biosensors for the nutritional environment. To explore this possibility, we examined, in parallel, the intracellular survival of additional *S. aureus* mutants identified in the Tn-seq screen, including *gudB* (SAUSA300_RS04645) and *lytR* (SAUSA300_RS01360) across all donors. Several genes within the *qox* operon were also unique to IL-10 conditions (*qoxB* and *qoxD*); therefore, a *qoxA* mutant (SAUSA300_RS05175) was examined since it is the first gene in the operon. Although MprF was important for *S. aureus* survival in HMDMs across all donors, differences were still evident where the *mprF* mutant was unable to persist in HMDMs from some individuals but was less affected in others (i.e., donors 2 and 5; Fig. 6B). Interestingly, the *qoxA* mutant displayed increased survival compared to WT bacteria in five of the six HMDM donors regardless of IL-10 exposure (Fig. 6B), suggesting that *qoxA* or other pathways influenced by the gene are deleterious to *S. aureus* intracellular survival. In contrast, no significant reductions in survival were observed with any of the other mutants tested compared to WT (Fig. 6B). Collectively, these findings demonstrate a high degree of donor variability in the HMDM metabolome, which may influence the pathways required for *S. aureus* intracellular survival in macrophages, although other mechanisms likely play a role.

## DISCUSSION

Here, we show that primary HMDMs are a nidus for *S. aureus* survival to accelerate biofilm formation. Since clinical *S. aureus* biofilm infections are initiated with a small inoculum, resident tissue macrophages may represent an important initial reservoir for infection. *S. aureus* intracellular replication would allow for a protected niche until a higher bacterial load is achieved, which may serve as a nucleation point for biofilm development. This intracellular lifestyle of *S. aureus* has been seen across a wide array of clinical isolates from different infection types (18), demonstrating that this could be an important aspect of clinical infection.

We leveraged Tn-seq to identify the strategies that *S. aureus* uses to survive within HMDMs and how this is impacted by IL-10. Our results suggest that IL-10 polarization expands the essential genome of *S. aureus* within the intracellular compartment. Although IL-10 treatment skews HMDMs to an anti-inflammatory state (60), the cytokine also elicits metabolic remodeling. Namely, IL-10 promotes oxidative metabolism in macrophages and our prior work has shown that *S. aureus* biofilm-derived lactate promotes IL-10 production by macrophages that is mediated by epigenetic remodeling (41) and biases macrophages toward OxPhos (38). Reprogramming macrophage metabolism using the OxPhos inhibitor oligomycin effectively skewed macrophages toward a proinflammatory state that was associated with an increase in intracellular glucose (38), indicative of a transition toward glycolysis, which is linked to proinflammatory activity. Therefore, the metabolic changes induced by IL-10 in HMDMs would be expected to attenuate antimicrobial pathways, such as ROS production, which might explain the accelerated biofilm formation observed in IL-10-treated HMDMs. Although exposure of HMDMs to IL-10 was found to increase biofilm thickness, no significant differences in *S. aureus* intracellular survival were observed between IL-10 and untreated macrophages. This may result from increased death of IL-10-treated HMDMs based on the known anti-inflammatory effects of the cytokine. Furthermore, antibiotics were not present during our HMDM-biofilm growth studies, such that heightened escape of bacteria from IL-10-treated HMDMs could account for the increased biofilm thickness observed. Nevertheless, it is important to note that biofilm growth was also significantly increased in response to unstimulated HMDMs, compared to bacteria alone, revealing an overall ability of *S. aureus* to exploit macrophages to promote biofilm development and formation of complex tower-like structures. From a clinical standpoint, many *S. aureus* biofilm infections arise from surgical site infections. Tissue injury during surgery causes the release of danger-associated molecular patterns that elicit wound healing responses, including IL-10 production. IL-10 action would program resident and tissue infiltrating macrophages toward an anti-inflammatory pro-reparative phenotype, which may provide an initial window for unchecked *S. aureus* growth to accelerate biofilm development as suggested by our findings. However, additional studies are required to assess this possibility and identify causative mechanisms. Another possibility is that the altered HMDM metabolome elicited by IL-10 places unique pressures on *S. aureus*, resulting in an expansion in the number of essential genes identified by Tn-seq. Indeed, several *S. aureus* metabolic pathways were significantly enriched in IL-10 or untreated HMDMs with some intriguing distinctions between biosynthetic vs catabolic reactions observed. In general, *S. aureus* genes associated with negative regulation of metabolic pathways, including macromolecule and RNA metabolism, were enriched in IL-10-polarized HMDMs, whereas *S. aureus* biosynthetic pathways were significantly higher in untreated HMDMs. We identified several genes that were essential for *S. aureus* intracellular survival within HMDMs and shared between the two treatment conditions, including *mprF* and *atpA/atpG*, which have been characterized in other reports (25, 42).

Prioritization of Tn-seq hits based on fold-changes during validation would have likely been misleading because the number of possible Tn insertion sites in genes is variable. For example, genes with a higher number of potential insertion sites may have an artificially inflated fold-change when compared to the pseudo data sets. Because of this, genes were selected for validation from significantly enriched pathways, with an emphasis on genes involved in metabolism. This included *gudB* (SAUSA300_RS04645), *qoxA* (SAUSA300_RS05175)*, nifA* (SAUSA300_RS09085)*, cydA* (SAUSA300_RS05305)*,* and *lytR* (SAUSA300_RS01360) since IL-10 polarization of HMDMs has been shown to affect cellular metabolism (60), in agreement with our findings. However, the identification of *S. aureus* genes that were essential for survival in IL-10-treated macrophages proved difficult to validate. This may be partially explained by false positives, which are known to occur with Tn-seq (54). It could also result from cross-complementation by different mutants in the Tn library that could mask fitness defects within macrophages or activation of compensatory networks to negate the action of essential genes. Another explanation may be donor variability of HMDMs, as others have reported (61–63). For example, IL-10Rα or IL10Rβ expression may differ between individuals, which would affect HMDM sensitivity to IL-10, which was not assessed in this study. The impact of inter-donor HMDM variability on *S. aureus* intracellular survival also manifested with an *mprF* mutant that was identified as essential across all donors. Namely, the *mprF* mutant was completely killed in HMDMs from some subjects but was less affected in others. This demonstrates that even a robust phenotype can show fluctuations across individuals, further highlighting the importance of biological diversity when studying human immune responses.

Indeed, donor variability has been shown to drastically alter the intracellular proliferation rate of *Cryptococcus neoformans* (62) as well as affect other HMDM phenotypes (61, 63). Therefore, we sought to understand the extent of donor variation on HMDM metabolism and responsiveness to IL-10. Although the abundance of certain metabolites was conserved across the six HMDM donors examined, where IL-10 affected glycolytic and TCA cycle intermediates, other metabolites including nucleotides, nucleotide derivatives, and select amino acids were variable. Since *S. aureus* can access the host cell cytoplasm during intracellular replication (22), the abundance of these different metabolites could influence the essentiality of genes and pathways *S. aureus* needs to survive and/or replicate. Supporting this, it has been shown that the levels of branched-chain amino acids and pyruvate are connected to the expression of *S. aureus* virulence genes (64, 65).

There are several limitations of this study, the first being that targeted metabolomics was utilized to explore the donor-dependent variability of HMDMs. This technique was used since it is more quantitative than untargeted metabolomics but is an underrepresentation of the breadth of metabolite changes that may occur across donors (66–68). Second, our Tn-seq experiment was performed using HMDMs from a single donor. Due to the variability across individuals that was recognized later in this study, future work could utilize HMDMs from pooled donors or the Tn-seq pipeline could be repeated with macrophages from different individuals to minimize the contribution of donor variability. Finally, this study utilized a Tn-seq library derived from one *S*. *aureus* clinical isolate, CA-MRSA USA300 LAC; therefore, examination of additional clinical strains will be critical to explore the conserved or strain-specific strategies that *S. aureus* utilizes to survive within HMDMs due to the large genetic variation across strains (69). For example, some groups using different *S. aureus* isolates reported survival in HMDMs for several days before inducing host cell lysis (40, 70), while other groups have shown survival kinetics similar to what was observed in this study (20, 22). The large number of genes identified by Tn-seq in this study precluded a comprehensive validation approach; therefore, the biological impact of additional hits remains to be determined in future work.

During the review of this manuscript, Lo et al. reported results from an *S. aureus* Tn-seq study that also found some degree of divergence between Tn-seq predictions and biological changes in *S. aureus* invasion of macrophages (71). However, this work was performed with a human macrophage cell line (THP-1), which does not capture the genetic diversity of primary macrophages recovered from different donors. This limitation was highlighted in a recent report showing that *S. aureus* superoxide dismutases (*sodA/M*) are important for intracellular survival in HMDMs but not THP-1 cells (72). Nevertheless, both our study and the study of Lo et al. identified *mprF* and *ltaA* as essential for macrophage intracellular survival, supporting the robustness of these hits.

Collectively, our results demonstrate that IL-10 polarization of HMDMs may create a unique intracellular environment for *S. aureus* survival that is influenced by host cell metabolism. It is also a cautionary tale that phenotypes in HMDMs should be validated across numerous donors to assess their strength and, by extension, role in the survival of *S. aureus* or any other pathogen during intracellular growth.

## MATERIALS AND METHODS

### Human monocyte-derived macrophages

Human monocytes were purified from the whole blood of de-identified healthy donors using counter-current centrifugal elutriation by the University of Nebraska Medical Center Elutriation Core Facility. Monocytes were cultured in Roswell Park Memorial Institute (RPMI)-1640 medium (Cytiva) supplemented with 10% fetal bovine serum (FBS), 1% L-glutamine (200 mM; Corning), 1% HEPES (Cytiva), 1% antibiotic-antimycotic solution (Cytiva, USA), 50 µM beta-mercaptoethanol, and 100 ng/mL human macrophage colony-stimulating factor (BioLegend) for 7 days at 37°C to promote macrophage maturation.

### Bacterial strains

The strain background used throughout most studies was a skin and soft tissue isolate, *S. aureus* USA300 LAC (73, 74). In some experiments, UAMS-1 (75), MW2 (76), and SA564 (77) were used to evaluate whether IL-10 influenced intracellular survival in HMDMs across different clinical isolates. The USA300 LAC Tn library was a kind gift from Dr. Anthony Richardson (48). Aliquots of the library were prepared by propagating for 10 h in tryptic soy broth (TSB) (10:1 flask:volume ratio, 250 rpm) and stored at −80°C for future use. Prior to each experiment, an aliquot of the library was gently thawed at 37°C for 30 min and diluted in RPMI-1640 medium for compatibility with HMDMs during the Tn-seq protocol. The mutants used in this study were acquired from the Nebraska Transposon Mutant Library (78). Mutants were selected using erythromycin (5 µg/mL) and confirmed with PCR using primers flanking the Tn insertion site (Table S1). To visualize bacteria for confocal microscopy, mutant strains were transduced with pCM29 (constitutive GFP expression) (79) using φ11 and selected using chloramphenicol (10 µg/mL).

### Confocal microscopy

HMDMs were stained with CellTracker Deep Red (1 µM; Invitrogen) and Hoechst 33342 (1 µg/mL; ImmunoChemistry Technologies) in 8-well glass-bottom chamber slides (Nunc) or 4-chamber 35 mm glass bottom dishes (Cellvis) and incubated with *S. aureus* JE-2 pCM29 (79) (constitutive GFP expression) or *S. aureus* JE-2 tdTomato (AH1263) (80) at an MOI of 10:1 (bacteria:macrophage). Samples were then visualized at the indicated time points using a Zeiss 710 META laser scanning confocal microscope with 40× oil magnification. Biomass, roughness coefficient, and average thickness measurements were determined using Comstat2 (ImageJ) (81, 82). For time-lapse microscopy, the stage and sample were maintained at 37°C with 5% CO_2_, and images were captured every 15 min over an 18-h interval.

### Gentamicin protection assay

HMDMs were treated with recombinant human IL-10 (100 ng/mL; BioLegend) or untreated for 16 h, then inoculated with WT and/or the indicated mutant *S. aureus* strains at an MOI of 10:1 (bacteria:macrophage) using a mono-infection or co-infection paradigm. After a 30-min phagocytosis period, HMDMs were treated with a high dose of gentamicin (100 µg/mL) for 30 min to kill remaining extracellular bacteria and then transitioned to a low gentamicin dose (1 µg/mL) to prevent intracellular accumulation of gentamicin that may occur with extended incubation periods. At the indicated time points, HMDMs were washed with phosphate-buffered saline (PBS), lysed using sterile water, and plated on trypticase soy agar (TSA) with 5% sheep blood to quantify intracellular bacterial burden. For co-infection experiments, dual plating on TSA ± erythromycin (5 µg/mL) was used since all *S. aureus* transposon mutants harbored an erythromycin resistance marker.

### Transposon sequencing

#### Sample collection

To determine the essential genes for *S. aureus* to survive within HMDMs, we used a modified version of the gentamicin protection assay described above to achieve sufficient intracellular bacteria for Tn-seq. Untreated or IL-10-treated HMDMs were inoculated with a saturated *S. aureus* Tn library at an MOI of 10:1. The phagocytosis period was extended to 2 h, whereupon HMDMs were treated with high dose gentamicin (100 µg/mL) for 30 min and then transitioned to the low gentamicin concentration (1 µg/mL) for 2 h. HMDMs were lysed with sterile water to recover viable intracellular bacteria, which were outgrown for 3 h in TSB (10:1 flask:volume ratio, 250 rpm, 37°C) to increase bacterial density for sequencing. Simultaneously, an aliquot of the same Tn-seq library used for HMDM infection was propagated in TSB for 3 h as an outgrowth control.

#### Sequencing and essential gene analysis

Following the 3-h outgrowth period, bacteria from each condition (outgrowth control, untreated HMDM, and IL-10-treated HMDM) were pelleted, DNA was extracted, and Tn-seq libraries were prepared as previously described (50). The libraries were sequenced on an Illumina NextSeq 500 using a 75 base pair single-end run. Reads were trimmed of adapter sequences using Cutadapt (83) and then mapped to the reference genome (*S. aureus* USA300_FPR3757) with Bowtie2 (84). The essential genome of each sample was then determined using a previously established, simulation-based Monte-Carlo method (50, 51) using DESeq2 (85). Genes were deemed essential if (i) the fold-change was significantly reduced compared to the expected data set (Benjamini-Hochberg adjusted *P* < 0.01; negative binomial Wald test in DESeq2) and (ii) clustered with the lower mode in the characteristic bimodal distribution of fold changes comparing the observed and expected data sets (mclust *P* < 0.01). Essential gene lists were then analyzed by a statistical over-representation test in PANTHERdb (www.PANTHERdb.org) (86, 87) using the GO Biological Process Complete annotation data set (88) to identify essential pathways of interest (86, 87, 89).

### Metabolomics

#### Sample preparation

Human monocytes from six different de-identified healthy donors were differentiated into HMDMs in 6-well tissue culture-coated dishes for 7 days, whereupon non-adherent cells were removed, and HMDMs were treated with recombinant human IL-10 (100 ng/mL) or left untreated for 16 h. Next, HMDMs were washed and incubated in medium alone [RPMI-1640, 10% FBS, 1% L-glutamine (200 mM), 1% HEPES buffer (1 M)] or stimulated with 10^6^ heat-killed *S. aureus* for 6 h. For metabolite extraction, HMDMs were washed 2× with PBS prior to the addition of pre-chilled (−80°C) 80% methanol and snap frozen at −80°C for 30 min. HMDMs were then scraped from the plate bottom on dry ice, collected into pre-chilled tubes, and centrifuged (14,000 rpm, 10 min). The supernatant was then transferred to a new tube, samples were dried in a SpeedVac (6.5 h, 30°C), and then held at −80°C until metabolomics analysis.

#### LC-MS/MS analysis of metabolites

Dried samples were reconstituted in 100 µL of 50% methanol. A triple-quadrupole-ion trap hybrid mass spectrometer (QTRAP6500+, Sciex, USA) connected to an ultra-performance liquid chromatography *I*-class system (Waters, USA) was used for metabolite analysis. Chromatographic separation was performed by liquid chromatography using an XBridge Amide (150 ×  2.1 mm ID; 3.5 µm particle size, Waters, USA) analytical column and a binary solvent system with a flow rate of 0.4  mL/min. A guard XBridge Amide column (20 ×  2.1 mm ID; 3.5 µm particle size, Waters) was connected before the analytical column. Mobile phase A was composed of 10 mM ammonium acetate and 10 mM ammonium hydroxide containing 5% acetonitrile in LC-MS grade water, whereas mobile phase B was 100% LC-MS grade acetonitrile. Columns were maintained at 40°C, and the autosampler temperature was maintained at 5°C. The injection volume of each sample was 5 µL, and a total of 1,000 µL of weak wash solvent comprising 10% aqueous methanol was used after each injection. The QTRAP6500 + instrument was operated in polarity switching mode for targeted quantitation of amino acids through a multiple reaction monitoring process. Electrospray ionization parameters were optimized as follows: electrospray ion voltage of −4,200 and 5,500 V in negative and positive modes, respectively; source temperature of 400°C, curtain gas of 35, and gas 1 and 2 of 40 and 40 psi, respectively. Compound-specific parameters were optimized for each compound using manual tuning. These parameters included declustering potential that was 65 and −60 V in the positive and negative modes, respectively; entrance potential that was set at 10 and −10 V in the positive and negative modes, respectively; and collision cell exit potential that was maintained at 10 and −10 V in the positive and negative modes, respectively.

#### Metabolomics analysis

For each metabolomics run, missing values were estimated by one-fifth of the minimum positive value of each metabolite, and concentrations were then log-transformed (base 2). Data from each batch were combined, and metabolites that were not detected in more than 50% of the samples were removed from downstream analysis. To correct for batch effects due to technical variation in sample preparation and/or MS analysis (90), DBnorm was used to compare a variety of statical methods for batch correction (59), and a *ber-bagging* model (58) was selected as it achieved the highest score for data correction on our data set. Following batch correction, the data were then imported into MetaboAnalyst version 5.0 (www.metaboanalyst.ca) (91) for further analysis including PCA.

### Statistics

Significant differences were determined using a two-way analysis of variance (ANOVA) with Sidak’s multiple comparisons test, one-way ANOVA with Dunnett’s multiple comparisons test, or unpaired Student’s *t*-test using GraphPad Prism version 9.0.2. *P* < 0.05 was considered statistically significant.

## Data Availability

Tn-seq and metabolomics data are provided in Data Sets S1 and S2, respectively, in the supplemental material. Raw transposon insertion sequencing files have been deposited in the NCBI Sequence Read Archive, https://www.ncbi.nlm.nih.gov/sra (BioProject accession number PRJNA1033637).

## SUPPLEMENTAL MATERIAL

Data Set S1 iai.00474-23-s0001.xlsx Tn-seq essential summary.

Data Set S2 iai.00474-23-s0002.xlsx Ber bagging corrected metabolomics.

Supplemental material iai.00474-23-s0003.pdf Fig. S1 to S5, Table S1, and legend for Video S1.

Video S1 iai.00474-23-s0004.mp4 *S. aureus* replication in human monocyte-derived macrophages (HMDMs).

ASM does not own the copyrights to Supplemental Material that may be linked to, or accessed through, an article. The authors have granted ASM a non-exclusive, world-wide license to publish the Supplemental Material files. Please contact the corresponding author directly for reuse.
