# Supplementary material for: Metabolic diversity of human macrophages: potential influence on Staphylococcus aureus intracellular survival
Source: Infect Immun. 2024 Jan 5;92(2):e00474-23. doi: 10.1128/iai.00474-23 (PMC10863412; doi:10.1128/iai.00474-23)
Supplement: Supplemental material — Fig. S1 to S5, Table S1, and legend for Video S1. [file iai.00474-23-s0003.pdf]

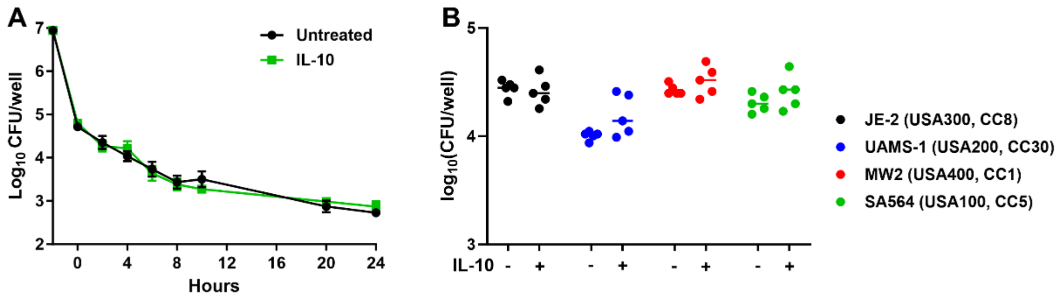

Figure S1. IL-10 does not affect *S. aureus* intracellular survival in human monocyte-derived macrophages (HMDMs). HMDMs were untreated or exposed to IL-10 for 16 h before challenge with (**A**) JE-2 (results representative of 5 independent experiments) or (**B**) various *S. aureus* strains at a MOI of 10:1 (bacteria:macrophage) to evaluate intracellular survival using gentamicin protection assays. HMDMs were lysed at the (**A**) indicated time points or (**B**) 24 h and CFUs were quantified.

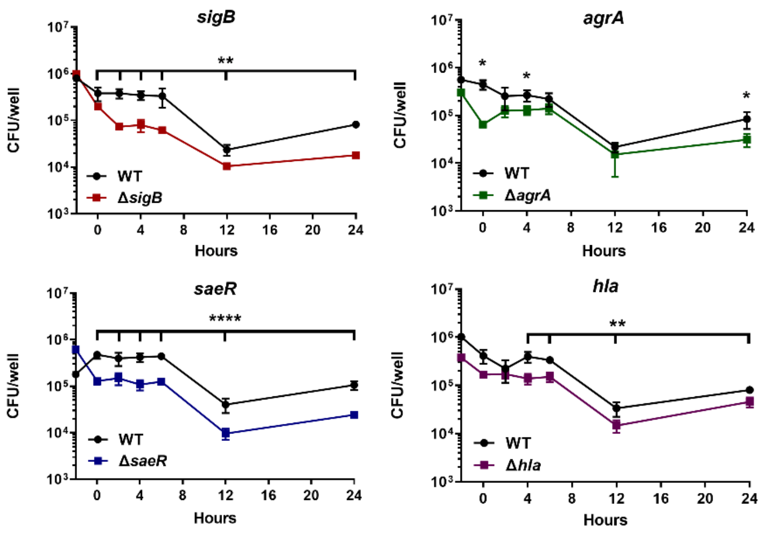

Figure S2. Intracellular survival of select *S. aureus* mutants in human monocyte-derived macrophages (HMDMs). HMDMs were exposed to WT *S. aureus* in competition with either Δ*sigB*, Δ*agrA*, Δ*saeR*, or Δ*hla* at a 1:1 ratio and a MOI of 10:1 (bacteria:macrophage) to evaluate intracellular survival using gentamicin protection assays. HMDMs were lysed at the indicated time points and CFUs were quantified by dual plating on trypticase soy agar ± erythromycin. (n = 4 from one independent experiment; *, p < 0.05; **, p < 0.01; ****, p < 0.0001; two-way ANOVA with Sidak's multiple comparisons test).

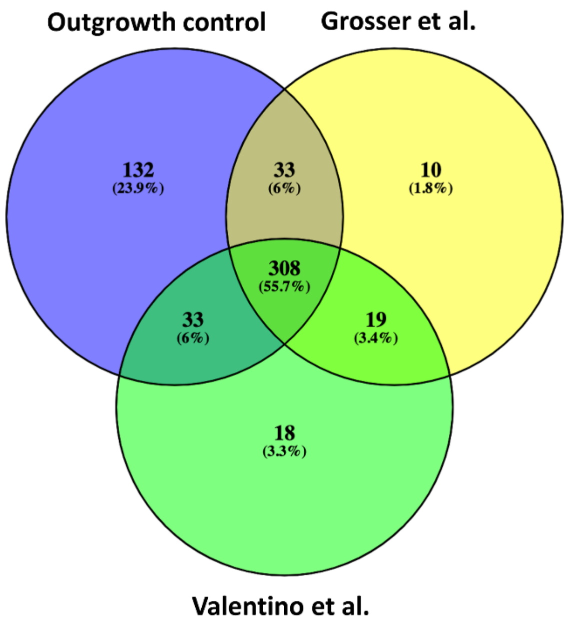

Figure S3. Essential genes from *S. aureus outgrowth* control overlap with previously published data sets. An aliquot of the *S. aureus* Tn library used in this study was propagated for 3 h as an outgrowth control. Essential genes from this sample were identified using a previously established Monte-Carlo method and compared with the controls from two published data sets to evaluate overlap (Grosser et al., https://doi.org/10.1371/journal.ppat.1006907; Valentino et al.; https://doi.org/10.1128/mBio.01729-14).

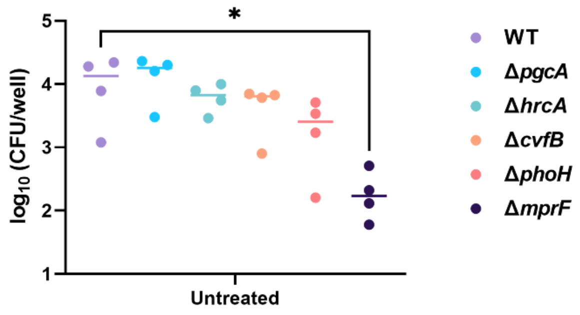

Figure S4. Intracellular survival of *S. aureus* mutants in human monocyte-derived macrophages (HMDMs). HMDMs were infected with *S. aureus* WT or select mutants at a MOI of 10:1 (bacteria:macrophage). After 24 h, HMDMs were lysed, and intracellular bacterial burden was quantified (n = 4 from one experiment; *, $p < 0.05$; one-way ANOVA with a Dunnett's multiple comparisons test against WT for each condition).

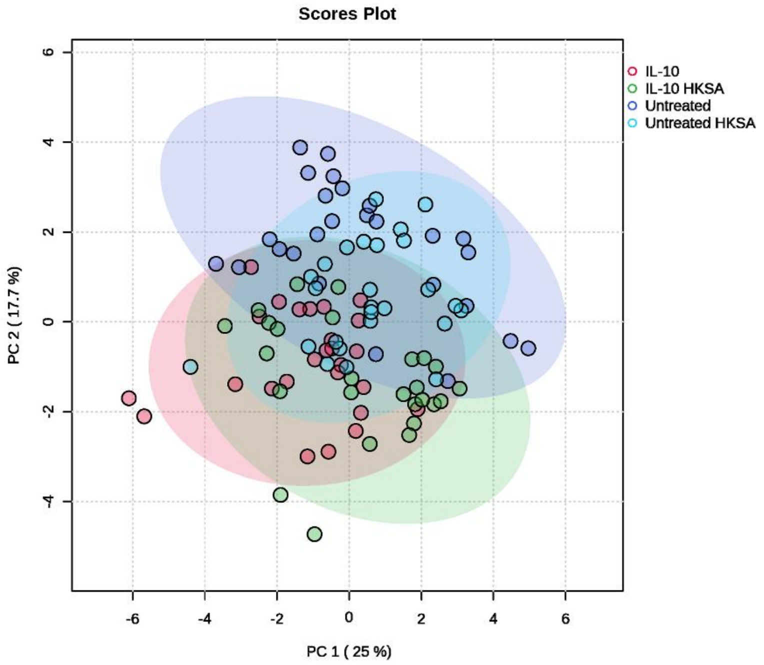

Figure S5. The metabolic profile of human monocyte-derived macrophages (HMDMs) in response to a proinflammatory stimulus. HMDMs were untreated or exposed to IL-10 for 16 h and then stimulated with either 10^6 heat-killed *S. aureus* (HKSA) or vehicle for an additional 6 h, whereupon intracellular metabolites were isolated and quantified by LC-MS/MS. Data from each donor was batch-corrected and analyzed using principal component analysis (PCA).

Table S1. Primers used in this study.

**qoxA_F**	TTTCAACGCAAAAGGGCCAG
**qoxA_R**	CGACCTCTGAACGTACCAGT
**nifA_F**	ATGACAACAATTAACCCAACAAACT
**nifA_R**	ATCGATCCGTTATAGGGGCA
**mprF_F**	CTGGTGGTTTCGGCGCTTTC
**mprF_R**	GGCGTTTCAACCTACGTGCT
**cydA_F**	GCTATGGCCAAAAGATGGGC
**cydA_R**	AATGCCATCGTCATACCGGC
**butA_F**	GAAGAAGGGGCAAAAGCAGC
**butA_R**	TTGTTCCCAACCCCATGCTT
**gudB_F**	TGCGTTTCCACCCAGATGTT
**gudB_R**	TGGATCGTGTAATGCACCGT
**lytR_F**	GAAACTGCAACACATGTACGTAT
**tn_R**	GCTTTTTCTAAATGTTTTTTAAGTAAATCAAGTAC

Video 1. *S. aureus* replication in human monocyte-derived macrophages (HMDMs). HMDMs were stained with CellTracker Deep Red (red) and challenged with live *S. aureus*-tdTomato (pseudo-colored green) at a MOI of 10:1 (bacteria:macrophage). Time lapse images were acquired every 15 min over an 18 h interval with cells incubated at 37°C with 5% CO_2. The arrow depicts a macrophage that lyses, releasing bacteria into the extracellular space.

Extended Dataset 1. Tn-seq essential summary

Extended Dataset 2. Ber bagging corrected metabolomics
